# Investigating Leggett-Garg inequality for a two level system under decoherence in a non-Markovian dephasing environment

**DOI:** 10.1038/srep06165

**Published:** 2014-08-22

**Authors:** Po-Wen Chen, Md. Manirul Ali

**Affiliations:** 1 Physics Division, Institute of Nuclear Energy Research, Longtan, Taoyuan 32546, Taiwan; 2 Department of Physics, National Cheng Kung University, Tainan 70101, Taiwan

## Abstract

Leggett-Garg inequalities (LGI) test the correlations of a single system measured at different times. Violation of LGI implies either the absence of a realistic description of the system or the impossibility of measuring the system without disturbing it. We investigate the violation of the Leggett-Garg inequality for a two level system under decoherence in a *non-Markovian* dephasing environment. We discuss the non-Markovian dynamics of the violation of LGI at zero temperature and also at finite temperature for different structured environments. An enhanced quantum coherence is shown through the violation of Leggett-Garg inequality in the strong non-Markovian regime of the environment.

There has been recent attention and renewed interests in the investigation of Leggett-Garg (LG) inequalities. The original motivation^1^ for these inequalities was to test quantum coherence in macroscopic systems. LG inequalities also play a useful role for microscopic systems as an indicator of nonclassicality due to the existence of superposition states and their collapse under measurement in the quantum regime. The LG inequalities were derived under following two assumptions (A1) *macrorealism*: performance of a measurement on a macroscopic system reveals a well-defined pre-existing value (A2) *noninvasive measurability*: this pre-existing value can be measured without disturbing the system. Violation of Leggett-Garg inequalities (LGI) implies either the absence of a realistic description of the system or the impossibility of measuring the system without disturbing it. Quantum mechanics (QM) violates the inequalities on both accounts. In recent years, theoretical study of QM violation of LGI has been carried out for a variety of systems like electron transport through quantum dot (to distinguish classical and quantum transport)^2^, for investigating nonclassicality in opto-electro-mechanical systems^3^, to investigate quantum coherence in oscillating neutral kaons^4^ and in biological light-harvesting protein complex^5^. The first measured violation of LGI was announced^6^ by Palacios-Laloy *et al.* and within a few years, violations had been probed in a diverse range of physical systems such as photons^7^,^8^,^9^, defect centres in diamond^10^, nuclear magnetic resonance^11^,^12^, phosphorus impurities in silicon^13^.

Violation of LGI is associated to the presence of coherence in the quantum system which inevitably interacts with its surrounding environment (reservoirs or baths) and the coherence is lost in general due to this unavoidable interaction. Generally, the non-unitary evolution of the reduced-density matrix of the system is obtained after taking partial trace of the total system-plus-reservoir density operator *ρ_T_*(*t*) over the reservoir degrees of freedom. Some approximations are often made in the derivation of master equation for the system's reduced density matrix. The most important approximations^14^,^15^,^16^ are the weak coupling or Born approximations, assuming that the coupling between the system and the reservoir is small enough to justify a perturbative approach, and the Markov approximation, assuming that the correlation time of the reservoir is very short compared to the typical system response time so that the reservoir correlation function is assumed to be *delta* correlated in time. Although, the use of the Markovian approximation is justified in a large variety of quantum optical experiments, one should notice that non-Markovian effects are crucial, for example, for high-speed quantum communication where the characteristic time of the relevant system becomes comparable with the reservoir correlation time, or if the environment is structured with a particular spectral density, for example, for quantum systems embedded in solid-state devices, where memory effects are typically non-negligible. In these cases, the dynamics can be substantially different from the Markovian dynamics. Due to their fundamental importance in quantum information processing and quantum computation, non-Markovian quantum decoherence have attracted much attention in recent years^18^,^19^,^20^,^21^,^22^,^23^,^24^,^25^,^26^, one of the main purposes of which in the long run is to engineer different types of (artificial) reservoirs and couple them to the system in a controlled way.

The evolution equation of an open system density matrix is governed by the reduced Liouville equation or called the quantum master equation that can be Markovian or non-Markovian. In the Markovian case, an useful procedure to calculate the two-time (multi-time) correlation functions (CFs) for open (dissipative) quantum systems is the so-called quantum regression theorem (QRT)^14^,^17^ that gives a direct relation between the time evolution equation of the single-time expectation values and that of their corresponding two-time (multi-time) CFs. So, knowing the master equation and the time evolution of the reduced density matrix, one is able to calculate the single-time expectation values as well as the two-time (multi-time) CFs using QRT. But knowing the time evolution of the reduced density matrix is not sufficient to calculate the temporal (two-time or multi-time) correlation function (CFs) of system observables in non-Markovian case, for non-Markovian open (dissipative) quantum systems, the QRT is not valid in general^37^,^38^,^39^,^40^. Leggett-Garg inequalities test the correlations of a single system measured at different times, so one needs to calculate these two-time correlation functions “〈*Q*(*t_j_*)*Q*(*t_i_*)〉” of an observable *Q* for this open quantum system. Two-time (multi-time) correlation functions (CFs) of an open quantum system in itself are important physical quantities^14^,^15^. A very useful evolution equation that allows systematically calculating the two-time correlation functions (CFs) of system operators for non-Markovian open (dissipative) quantum systems is derived^40^ recently. The derivation is based on perturbative quantum master equation approach, valid to second order in the system-environment interaction. We will use this method to calculate the two-time correlation functions for a two level system in a structured non-Markovian dephasing environment, and finally investigate the non-Markovian dynamics of LGI-violation (coherence) for this specific open quantum system. The two-time CFs obtained using the derived evolution equation^40^,^41^ in the weak system-environment coupling case for this non-Markovian pure-dephasing model happen to be the same as those obtained from the exact evaluation. However, the two-time correlation functions so obtained significantly differ from the non-Markovian two-time CFs obtained by wrongly directly applying the quantum regression theorem (QRT), a useful procedure to calculate the two-time CFs for weak-coupling Markovian open systems.

The simplest LGI can be constructed as follows. Let *Q*(*t*) be the observable of a two level system such that, whenever measured, it is found to take a value +1 or −1, depending on whether the system is in state |+〉 or |−〉. We then perform three set of experimental runs such that in the first set of runs *Q* is measured at times *t*_1_ and *t*_2_ = *t*_1_ + Δ*t*; in the second, at *t*_1_ and *t*_3_ = *t*_1_ + 2Δ*t*; in the third at *t*_2_ and *t*_3_ (here *t*_3_ > *t*_2_ > *t*_1_). From such measurements, it is intuitive to determine the two-time correlation functions *K_ji_* = 〈*Q*(*t_j_*)*Q*(*t_i_*)〉 where *t_j_* > *t_i_*. Leggett and Garg^1^ adopted the standard argument leading to a Bell-type inequality, with times *t_i_* and *t_j_* playing the role of apparatus settings. For any set of runs corresponding to the same initial state, any individual *Q*(*t*) has the same definite value (*assumption* A1), irrespective of the pair *Q*(*t_j_*)*Q*(*t_i_*) in which it is measured; i.e., the value of *Q*(*t_j_*) or *Q*(*t_i_*) in any pair does not depend (*assumption* A2) on whether any prior or subsequent measurement has been made on the system. Consequently the combination *Q*(*t*_2_)*Q*(*t*_1_) + *Q*(*t*_3_)*Q*(*t*_2_) − *Q*(*t*_3_)*Q*(*t*_1_) has an upper bound of +1 and lower bound of −3. If all the individual product terms in this expression are replaced by their averages over the entire ensemble of such sets of runs, the following form of LGI is then obtained: 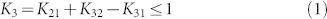
The same LGI can also be derived through probability argument^42^. With similar arguments one can also derive a Leggett-Garg inequality for measurements at four different times, *t*_1_, *t*_2_, *t*_3_ and *t*_4_ = *t*_1_ + 3Δ*t* given by 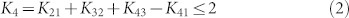
The two-time operators *Q*(*t_j_*)*Q*(*t_i_*) are not Hermitian operators, and the two-time correlation functions *K_ji_* = 〈*Q*(*t_j_*)*Q*(*t_i_*)〉 are in general complex quantities. We consider the symmetrised combination of the two-time correlators: *K_ji_* = 〈{*Q*(*t_j_*), *Q*(*t_i_*)}〉/2, to indentify them with physical expectation values of the two-time measurements. Please note that the symmetrised operator 〈{*Q*(*t_j_*), *Q*(*t_i_*)}〉/2 = (*Q*(*t_j_*)*Q*(*t_i_*) + *Q*(*t_i_*)*Q*(*t_j_*))/2 is Hermitian whose expectation value can be associated to the average value of the two-time measurements (see Supplementary Information and^43^). Hence, we will only consider the real part of *K*_3_ and *K*_4_ to investigate the Leggett-Garg inequalities for a two level system interacting with a non-Markovian dephasing environment. Recently, a general framework for understanding the influence of non-unitary evolution on maximal violations of the LGI was given^44^ for a class of Markovian decoherence channels. Using LG inequalities, another proposal^45^ was given to test quantum coherent dynamics of a two-qubit system undergoing through a superradiant decay under a common reservoir, Born-Markov master equation was used for the open system dynamics. For the experimental realization, they provided a feasible scheme, which consists of two quantum dots coupled to nanowire surface plasmons. Dynamics of the violation of the Leggett-Garg inequality for a two level system under decoherence in a *non-Markovian* dephasing environment, to our knowledge, have not been presented in the literature. We will briefly discuss the pure-dephasing spin-boson model and the perturbative method to calculate the non-Markovian evolution equation for reduced density matrix and two-time CFs valid up to second order in system-environment coupling strength. Then we present our numerical results to investigate Leggett-Garg inequality under the non-Markovian decoherence dynamics, where we show an enhanced quantum coherence through the violation of Leggett-Garg inequality in a suitably tuned non-Markovian regime of the environment.

## Results

First, we discuss on the evolution equation of non-Markovian two-time correlation functions. We consider a general class of open quantum systems modeled by the total Hamiltonian of the system plus reservoir as 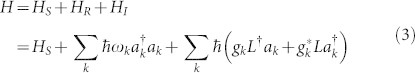
where *H_S_* and *H_R_* are system and environment Hamiltonian, respectively, and *H_I_* describes the interaction between the system and the environment. Here the operator *L* acts on the Hilbert space of the system, 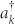 and *a_k_* are the creation and the annihilation operators on the bosonic environment Hilbert space, and *g_k_* and *ω_k_* are the coupling strength and the frequency of the *k*th environmental oscillator, respectively. The reduced density operator of the system can be obtained from the density operator of the total system by tracing over the environmental degrees of freedom *ρ_S_*(*t*) = Tr*_E_* (*ρ_T_*(*t*)). The total density operator is governed by the quantum evolution: 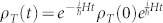. One can obtain a time-convolutionless non-Markovian master equation at finite temperature, valid to second order in the system-environment interaction strength 
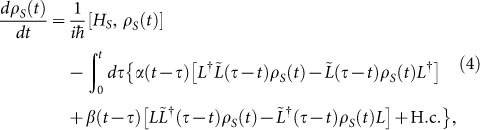
where 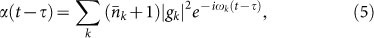

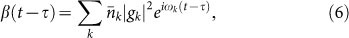
are the environment CFs with 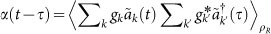 and 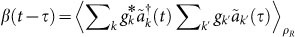, where 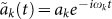 and 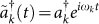 are the environment operators in the interaction picture, and the symbol 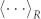 denotes taking a trace with respect to the density matrix of the thermal reservoir (environment). Here 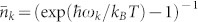 is the thermal mean occupation number of the bosonic environment oscillators.

**Figure 1:**
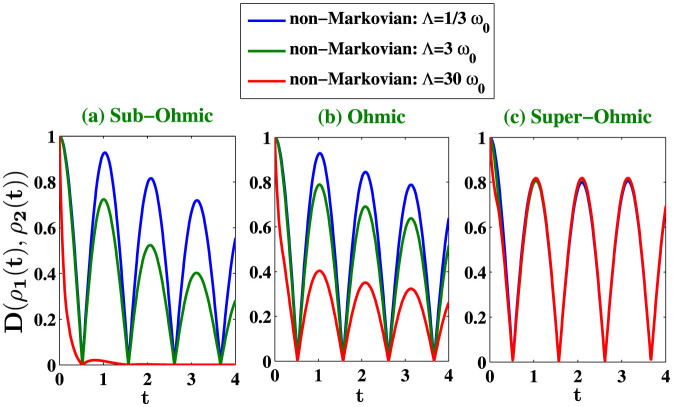
We plot the trace distance *D*(*ρ*_1_(*t*), *ρ*_2_(*t*)) at zero temperature for three different values of *s* = 0.5, *s* = 1, *s* = 3 corresponding to sub-Ohmic, Ohmic and super-Ohmic environments with *γ* = 0.05. For each environment, we consider three different cutoff frequencies Λ = *ω*_0_/3, Λ = *ω*_0_, and Λ = 30*ω*_0_.

**Figure 2:**
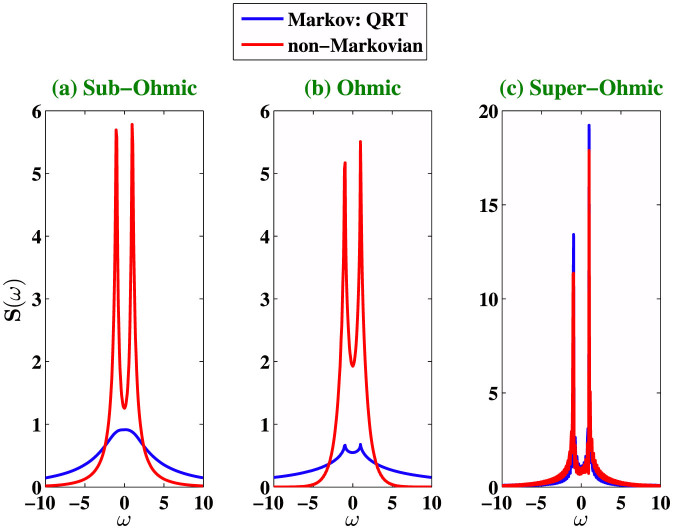
We plot the noise spectrum *S*(*ω*) at zero temperature for three different values of *s* = 0.5, *s* = 1, *s* = 3 corresponding to sub-Ohmic, Ohmic and super-Ohmic environments with cutoff frequency Λ = *ω*_0_/3, coupling strengths *γ* = 0.05 and *t* = 0.1. We compare the non-Markovian noise power spectrum *S*(*ω*) with its Markovian counterpart calculated by using the quantum regression theorem (QRT).

**Figure 3:**
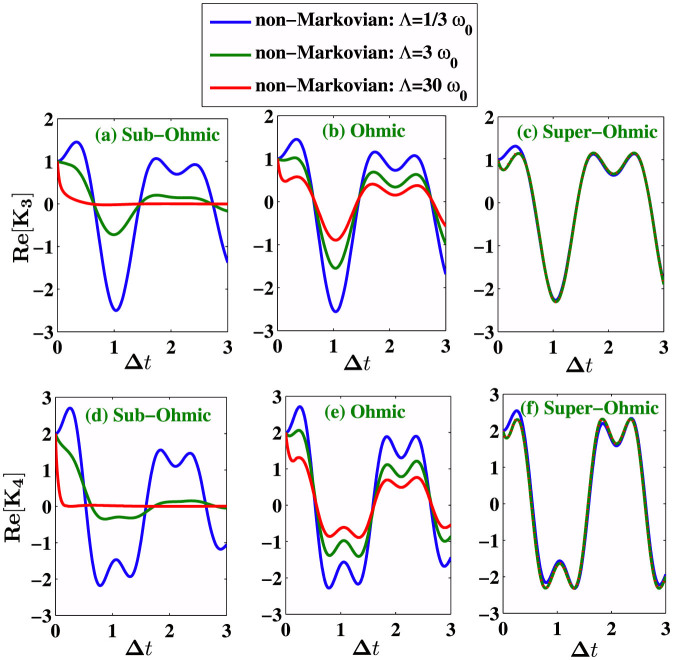
We plot real parts of *K*_3_ and *K*_4_ at zero temperature for three different values of *s* = 0.5, *s* = 1, *s* = 3 corresponding to sub-Ohmic, Ohmic and super-Ohmic environments with *γ* = 0.05. For each environment, we consider three different cutoff frequencies Λ = *ω*_0_/3, Λ = *ω*_0_, and Λ = 30*ω*_0_.

**Figure 4:**
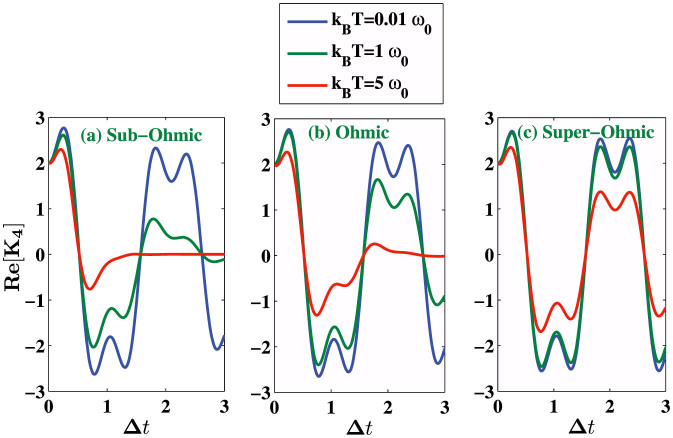
We plot real part of *K*_4_ at finite temperature for three different values of *s* = 0.5, *s* = 1, *s* = 3 corresponding to sub-Ohmic, Ohmic and super-Ohmic environments with *γ* = 0.05 and Λ = *ω*_0_. For each environment, we consider three different temperatures *k_B_T* = 0.01*ω*_0_, *k_B_T* = *ω*_0_, and *k_B_T* = 5*ω*_0_.

**Figure 5:**
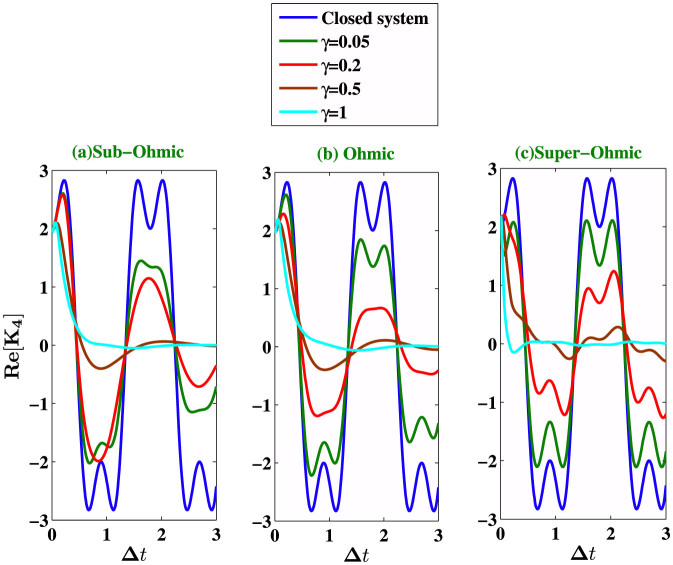
We compare the open system LGI violations with that corresponding to close system case. We plot the real part of *K*_4_ for the closed system and that in presence of the reservoir at zero temperature. We consider three different values of *s* (*s* = 0.5, *s* = 1, *s* = 3) corresponding to sub-Ohmic, Ohmic and super-Ohmic environments with cutoff frequency Λ = *ω*_0_/3. We consider four different values of the coupling strengths *γ* = 0.05, 0.2, 0.5, 1.0.

Using open quantum system technique and quantum master equation approach, one can also calculate the non-Markovian evolution equation of the two-time CFs of system observables *Q*(*t_i_*) and *Q*(*t_j_*) (for finite temperature environment), valid to second order in system-environment coupling as^40^

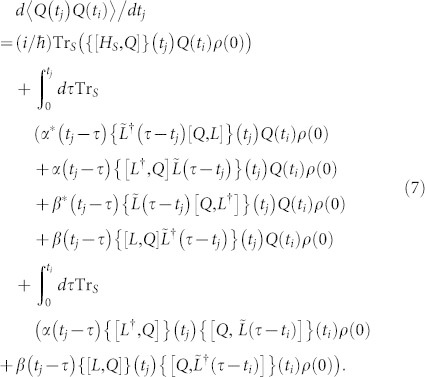
where 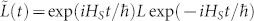 is the system operator of the interaction Hamiltonian (Eq. 3) in the interaction picture with respect to *H_S_* and *Q* = *Q*(0). In the derivation of Eqs.(4) and (7), we have considered the factorized initial system-bath state 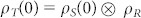, where *ρ_R_* = exp(−*βH_R_*)/*Tr*[exp(−*βH_R_*)]. Quantum regression theorem for this non-Markovian evolution is not applicable due to the presence of the last integral term in Eq.(7). For pure dephasing spin-boson model *L* = *σ_z_* = *L*^†^, and 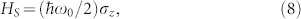
then the non-Markovian master equation for the reduced system density matrix becomes^16^

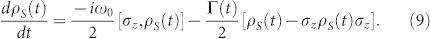
The non-Markovian effect in the master equation (9) is taken into account by the time-dependent coefficient Γ(*t*) given by 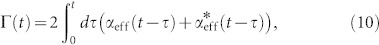
where *α*_eff_(*t* − *τ*) is the effective bath CF given by^41^

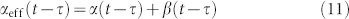
where *α*(*t* − *τ*) and *β*(*t* − *τ*) are defined in Eqs.(5) and (6). The single-time expectation value of an operator can be obtained as 
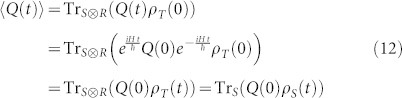
Using Eq.(7), one can also calculate the non-Markovian evolution equation of the two-time CFs of system observables as 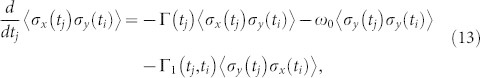

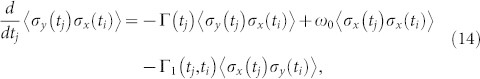

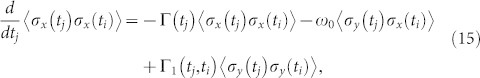

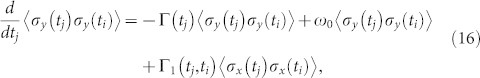
where 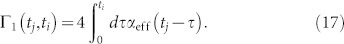
To calculate the functions Γ(*t*) and Γ_1_(*t_j_*, *t_i_*), we need to evaluate the environment correlation function: 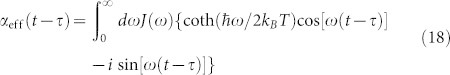
where *J*(*ω*) = Σ*_k_*|*g_k_*|^2^*δ*(*ω* − *ω_k_*) is the spectral density of the environment. We may consider any spectral density to characterize the environment, here we consider the following spectral density 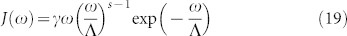
where *γ* is the coupling strength between the system and the environment and Λ is the cutoff frequency. When *s* < 1, 1, and >1, the corresponding environments are sub-Ohmic, Ohmic, and super-Ohmic respectively. Let us now discuss the non-Markovian characteristics of these environments through the dynamics given by Eq.(9). Recently important steps towards the development of a general consistent theory of non-Markovian quantum dynamics have been made and several measures have been proposed^27^,^28^,^29^,^30^,^31^,^32^,^33^ which try to define the border between Markovian and non-Markovian quantum evolution and to quantify memory effects in the open system dynamics. It is important to mention here that non-Markovianity can be used as a resource in the new quantum technologies. One can induce non-Markovianity, in the spirit of reservoir engineering, to improve quantum protocols such as quantum metrology and quantum key distribution^34^,^35^,^36^. Here, we consider a particular approach to demonstrate the non-Markovian dynamics of the two level system, based on the trace distance between quantum states^28^. Quantum memory effects can be visualized through the dynamics of the trace distance *D*(*ρ*_1_, *ρ*_2_) = (1/2) Tr |*ρ*_1_ − *ρ*_2_| between two quantum states *ρ*_1_ and *ρ*_2_. This quantity can be interpreted as a measure for the distinguishability of the two states. Markovian processes tend to continuously reduce the distinguishability of physical states, which means that there is a one way flow of information from the open system to its environment. In order to have non-Markovian effects, there must be, for some interval of time, an information flow from the environment back to the system. In view of this interpretation the characteristic feature of a non-Markovian quantum process is the increase of the distinguishability, that is a reversed flow of information from the environment back to the open system. The information flowing from the environment back to the system allows the earlier states of the system to have an effect on the later dynamics of the system, that is, it allows the emergence of memory effects. The non-Markovian dynamics of the two-level system is illustrated in Fig. 1 where we plot the trace distance *D*(*ρ*_1_(*t*), *ρ*_2_(*t*)) for the following pair of initial states 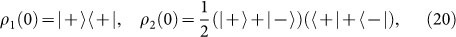
where where |+〉 and |−〉 are the eigenstates of *σ_z_*. Fig. 1 shows that the non-Markovian memory effect is enhanced for suitable choices of the cutoff frequencies for Sub-Ohmic and Ohmic environment, whereas the strong memory effect under a super-Ohmic environment is less sensitive to the cutoff frequency. We plot in Fig. 2, the noise power spectrum *S*(*ω*) for different type of environments. *S*(*ω*) is just the Fourier spectrum of 〈*σ_x_*(*t* + *τ*)*σ_x_*(*t*)〉 calculated using Eqs.(13–16). We compare the non-Markovian noise power spectrum *S*(*ω*) with its Markovian counterpart calculated by using the quantum regression theorem (QRT). The coherent peaks of the Fourier spectrum are higher and widths are narrower in the non-Markovian evolution. It is important to mention here that the noise power spectrum *S*(*ω*) was directly measured in the first experimental test of LGI violation^6^, and two-time correlation functions were then computed by inverse Fourier transformation of *S*(*ω*). Next, we investigate the dynamics of LGI for a two-level system under decoherence in a non-Markovian dephasing environment with *Q* = *σ_x_* and *K_ji_* = 〈*σ_x_*(*t_j_*)*σ_x_*(*t_i_*)〉 using Eq.(7). We stress that the non-Markovian effects are crucial for open quantum systems interacting with an environment structured with a particular spectral density. We show that the violation of LGI, or in other words the quantum coherence dynamics can be controlled with suitable choices of the environment spectral density. The initial environment state is considered to be in the thermal equilibrium state and the system is arbitrarily chosen to 
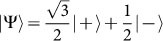
, hence *ρ_S_*(0) = |Ψ〉 〈Ψ|. Then we calculate *K*_3_ and *K*_4_ in three different cases with different structured environments characterized by the spectral density (19). In Fig. 3, we plot the real part of *K*_3_ and *K*_4_ for different values of the environmental parameter *s*. We show the dynamics of this inequality for different structured environments by varying the cutoff frequency Λ. In Figs. 3(a) and (d), we consider a sub-Ohmic (*s* = 0.5) zero temperature environment. In Figs. 3(b) and (e), we consider vacuum reservoir with Ohmic (*s* = 1) spectrum. Figs. 3(c) and (f) show the dynamics of *K*_3_ and *K*_4_ for a super-Ohmic (*s* = 3) vacuum bath. In general, the system dynamics goes beyond classical description (violation of LGI) for short measurement intervals Δ*t*. From Figs. 3(a) and (d), we notice that the quantities *K*_3_ and *K*_4_ are oscillatory and violate the LG inequalities with the strongest violation occurring at the first maxima, at 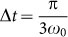, with cut-off frequency Λ = *ω*_0_/3. The maximum QM value of *K*_3_ or *K*_4_ depend on the value of the cut-off frequency Λ of the spectral density. We observe an enhanced dynamics of quantum violation of LGI (*K*_3_ > 1 and *K*_4_ > 2) at lower cut-off frequencies for sub-Ohmic and Ohmic environment. The enhanced quantum coherence dynamics is due to the stronger non-Markovian memory effect when the cut-off frequency is small. For sub-Ohmic and Ohmic spectrum, we see a reduced violation of LGI (Figs. 3(a), (d) and (b), (e)) as we increase the cut-off frequency. Fig. 3(c) and (f) indicate that the LGI violation is less sensitive to cutoff frequency in case of super-Ohmic (*s* = 3) environment, and we have wide range of Δ*t* where LGI is violated in case of super-Ohmic spectrum. This is also clear from Fig. 1, where we see an enhanced non-Markovian memory effect for Sub-Ohmic and Ohmic environment when the cut-off frequency is small, also the trace distance *D*(*ρ*_1_(*t*), *ρ*_2_(*t*)) is less sensitive to the cutoff frequency in case of super-Ohmic environment. Next, we go to the finite-temperature case for which 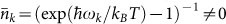. In Fig. 4, we show the effect of temperature on LG inequality for the system arbitrarily chosen in an initial state |Ψ〉 and the environment initially in the thermal equilibrium state. We calculate *K*_3_ and *K*_4_ for three different values of the environmental parameters “s” with fixed value of Λ = *ω*_0_. We find that at low temperature (*k_B_T* = 0.01*ω*_0_), revival of the violation of LGI occur, but at higher temperature (*k_B_T* = 5*ω*_0_) we see only one-peak violating the LGI (for very short measurement interval Δ*t*), and the system generally goes to classical regime at higher temperatures. Fig. 5 describes an explicit comparison between the LGI violation for the open system dynamics and the close dynamics (in absence of the environment) under the Hamiltonian (8). Two-time correlation functions for the closed system are calculated following the calculation in the supplementary material. We see that the LGI violation is reduced when the coupling strength *γ* of the two level system with its environment is gradually increased.

## Discussion

Non-Markovian quantum decoherence dynamics have attracted much attention in recent years^18^,^19^,^20^,^21^,^22^,^23^,^24^,^25^,^26^,^27^,^28^,^29^,^30^,^31^,^32^,^33^,^34^,^35^,^36^ with interesting memory effects, *e.g.*, non-divisibility of dynamical maps, back flow of information, dissipative dynamics with negative decay rate, non-monotonous increase of entropy of an open system, breakdown of quantum regression theorem etc., they all have fundamental importance in quantum information processing and quantum computation. We investigate the dynamics of quantum coherence through LG inequalities for a two-level system under decoherence in a non-Markovian dephasing environment where we show the dynamics of the violation of LGI at zero or finite temperature for structured environments with different spectral densities (19). We have utilized an useful method^40^ to calculate the dynamics of two-time correlation function and the noise spectrum for non-Markovian open quantum systems. An enhanced violation of LGI is observed in the strong non-Markovian regime when we lower the cutoff frequency of the environment. It is important to understand that how does the memory (non-Markovianity) of the environment quantitatively affect the violation of LGI. Generally, the non-Markovian nature of the system appears when the system gets correlated with the environment, so it is not *obvious* how does this system-environment correlation is related with the measurement correlation when the system is measured at different times. Our aim is to quantitatively address this question which is missing in the literature.

## Methods

The two-time correlation functions 〈*Q*(*t_j_*)*Q*(*t_i_*)〉 are calculated by solving the linear coupled differential equations (Eqs.13–16) with the initial values are given by following single-time expectation values 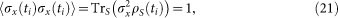

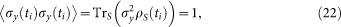

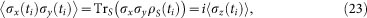

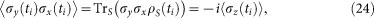
which are calculated using Eq.(12). The trace distance is defined as 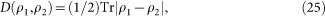
where the modulus of an operator *A* is defined by 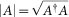.

## Author Contributions

Both the authors P.-W.C. and M.M.A. have made a significant contribution to the concept, calculation and interpretation of the present work.

## Supplementary Material

Supplementary Information Supplementary Information
