# Supplementary material for: Investigating Leggett-Garg inequality for a two level system under decoherence in a non-Markovian dephasing environment
Source: Sci Rep. 2014 Aug 22;4:6165. doi: 10.1038/srep06165 (PMC4141272; doi:10.1038/srep06165)
Supplement: Supplementary Information [file srep06165-s1.pdf]

Investigating Leggett-Garg inequality for a two level system under decoherence in a non-Markovian dephasing environment

Po-Wen Chen[1,*] and Md. Manirul Ali[2,†]

[1]*Division of Physics, Institute of Nuclear Energy Research, Taoyuan County 32546, Taiwan*

[2]*Department of Physics, National Cheng Kung University, Tainan 70101, Taiwan*

[*]powenchen69@gmail.com

[†]mani@mail.ncku.edu.tw

Supplementary Material

The symmetrised operator $\langle\{Q(t_j),Q(t_i)\}\rangle/2 = \left(Q(t_j)Q(t_i)+Q(t_i)Q(t_j)\right)/2$ is Hermitian whose expectation value can be associated to the average value of the two-time measurements

Let us consider a two level closed system evolving under the Hamiltonian $\mathcal{H} = \frac{\hbar}{2}\omega\sigma_z$ with an initial state $|\Psi(t_0)\rangle = \cos(\theta/2)|+\rangle + \sin(\theta/2)e^{i\phi}|-\rangle$, where $|+\rangle$ and $|-\rangle$ are the eigenstates of σ_z . We then perform an experiment where $Q = \sigma_x$ is measured at two different times, first at time $t = t_i$, then leaving the system undisturbed (allowing only unitary evolution under $\mathcal{H}$) until $t = t_j$ when σ_x is again measured. The eigenstates of σ_x are $|+\rangle_x$ and $|-\rangle_x$, and also we have considered $t_j > t_i$. There are four possible outcomes of this measurement (1) $\sigma_x(t_i) = +1$, $\sigma_x(t_j) = +1$ (2) $\sigma_x(t_i) = -1$, $\sigma_x(t_j) = +1$ (3) $\sigma_x(t_i) = +1$, $\sigma_x(t_j) = -1$ (4) $\sigma_x(t_i) = -1$, $\sigma_x(t_j) = -1$.

The probabilities of these outcomes are given by Bayes' theorem of probability

$$p_1 = p_{11}\left(\sigma_x(t_i) = +1\right)\ p_{12}\left(\frac{\sigma_x(t_j) = +1}{\sigma_x(t_i) = +1}\right) \tag{1}$$

$$p_2 = p_{21}\left(\sigma_x(t_i) = +1\right)\ p_{22}\left(\frac{\sigma_x(t_j) = -1}{\sigma_x(t_i) = +1}\right) \tag{2}$$

$$p_3 = p_{31}\left(\sigma_x(t_i) = -1\right)\ p_{32}\left(\frac{\sigma_x(t_j) = +1}{\sigma_x(t_i) = -1}\right) \tag{3}$$

$$p_4 = p_{41}\left(\sigma_x(t_i) = -1\right)\ p_{42}\left(\frac{\sigma_x(t_j) = -1}{\sigma_x(t_i) = -1}\right) \tag{4}$$

where $p_{11}\left(\sigma_x(t_i) = +1\right)$ is the probability to find the system in $|+\rangle_x$ when σ_x is measured first at $t = t_i$, and $p_{12}\left(\frac{\sigma_x(t_j)=+1}{\sigma_x(t_i)=+1}\right)$ is the probability to find the system in $|+\rangle_x$ after second σ_x measurement at $t = t_j$ with the condition that the system was projected into $|+\rangle_x$ after the first σ_x measurement at $t = t_i$. Other probabilities are defined accordingly.

To obtain p_1, we need to calculate both $p_{11}\left(\sigma_x(t_i) = +1\right)$ and the conditional probability $p_{12}\left(\frac{\sigma_x(t_j)=+1}{\sigma_x(t_i)=+1}\right)$. We start with the initial state

$$|\Psi(t_0)\rangle = \cos(\theta/2)|+\rangle + \sin(\theta/2)e^{i\phi}|-\rangle \tag{5}$$

The time evolved state at $t = t_i$ is given by

$$|\Psi(t_i)\rangle = \exp\left(-i\mathcal{H}(t_i - t_0)/\hbar\right) = \alpha_1|+\rangle_x + \beta_1|-\rangle_x, \tag{6}$$

where (for $t_0 = 0$)

$$\alpha_1 = \frac{1}{\sqrt{2}}\left[\cos(\theta/2)e^{-\frac{i}{2}\omega t_i} + \sin(\theta/2)e^{i\phi}e^{\frac{i}{2}\omega t_i}\right] \tag{7}$$

$$\beta_1 = \frac{1}{\sqrt{2}}\left[\cos(\theta/2)e^{-\frac{i}{2}\omega t_i} - \sin(\theta/2)e^{i\phi}e^{\frac{i}{2}\omega t_i}\right] \tag{8}$$

Then the probability to find the system in $|+\rangle_x$ when σ_x is measured at $t = t_i$ is given by

$$p_{11}\left(\sigma_x(t_i) = +1\right) = |\alpha_1|^2 = \frac{1}{2}\left[1 + \sin(\theta)\cos(\phi + \omega t_i)\right] \tag{9}$$

Next, we proceed to calculate the conditional probability $p_{12}\left(\frac{\sigma_x(t_j)=+1}{\sigma_x(t_i)=+1}\right)$. After we get the outcome $\sigma_x = +1$ at $t = t_i$, the state is collapsed to $|+\rangle_x$. We then allow the system to evolve from $t = t_i$ to $t = t_j$ under $\mathcal{H}$. The time evolved state at $t = t_j$ is given by

$$|\Psi(t_j)\rangle = \exp\left(-i\mathcal{H}(t_j - t_i)/\hbar\right)|+\rangle_x = \alpha_2|+\rangle_x + \beta_2|-\rangle_x, \tag{10}$$

where (for $t_j - t_i = \Delta t$)

$$\alpha_2 = \frac{1}{2}\left(e^{-\frac{i}{2}\omega\Delta t} + e^{\frac{i}{2}\omega\Delta t}\right) = \cos(\frac{\omega\Delta t}{2}) \tag{11}$$

$$\beta_2 = \frac{1}{2}\left(e^{-\frac{i}{2}\omega\Delta t} - e^{\frac{i}{2}\omega\Delta t}\right) = -i\sin(\frac{\omega\Delta t}{2}) \tag{12}$$

Then the probability to find the system in $|+\rangle_x$ after second σ_x measurement at $t = t_j$ (with the condition that the system was found in $|+\rangle_x$ after the first σ_x measurement at $t = t_i$) is

$$p_{12}\left(\frac{\sigma_x(t_j) = +1}{\sigma_x(t_i) = +1}\right) = |\alpha_2|^2 = \cos^2\left(\frac{\omega\Delta t}{2}\right) \tag{13}$$

Following similar procedure one can calculate all other probabilities

$$p_{21}\left(\sigma_x(t_i) = +1\right) = |\alpha_1|^2 = \frac{1}{2}\left[1 + \sin(\theta)\cos(\phi + \omega t_i)\right] \tag{14}$$

$$p_{31}\left(\sigma_x(t_i) = -1\right) = |\beta_1|^2 = \frac{1}{2}\left[1 - \sin(\theta)\cos(\phi + \omega t_i)\right] \tag{15}$$

$$p_{41}\left(\sigma_x(t_i) = -1\right) = |\beta_1|^2 = \frac{1}{2}\left[1 - \sin(\theta)\cos(\phi + \omega t_i)\right] \tag{16}$$

$$p_{22}\left(\frac{\sigma_x(t_j) = -1}{\sigma_x(t_i) = +1}\right) = |\beta_2|^2 = \sin^2\left(\frac{\omega\Delta t}{2}\right) \tag{17}$$

$$p_{32}\left(\frac{\sigma_x(t_j) = +1}{\sigma_x(t_i) = -1}\right) = |\beta_2|^2 = \sin^2\left(\frac{\omega\Delta t}{2}\right) \tag{18}$$

$$p_{42}\left(\frac{\sigma_x(t_j) = -1}{\sigma_x(t_i) = -1}\right) = |\alpha_2|^2 = \cos^2\left(\frac{\omega\Delta t}{2}\right) \tag{19}$$

Finally, the expectation or average value of this two-time measurements is given by

$$
\begin{aligned}
A &= (+1)p_1 + (-1)p_2 + (-1)p_3 + (+1)p_4 && (20)\\
&= (+1)(p_{11}p_{12}) + (-1)(p_{21}p_{22}) + (-1)(p_{31}p_{32}) + (+1)(p_{41}p_{42}) && (21)\\
&= (+1)\frac{1}{2}\left[1+\sin(\theta)\cos(\phi+\omega t_i)\right]\cos^2\left(\frac{\omega\Delta t}{2}\right) + \\
&\quad (-1)\frac{1}{2}\left[1+\sin(\theta)\cos(\phi+\omega t_i)\right]\sin^2\left(\frac{\omega\Delta t}{2}\right) + \\
&\quad (-1)\frac{1}{2}\left[1-\sin(\theta)\cos(\phi+\omega t_i)\right]\sin^2\left(\frac{\omega\Delta t}{2}\right) + \\
&\quad (+1)\frac{1}{2}\left[1-\sin(\theta)\cos(\phi+\omega t_i)\right]\cos^2\left(\frac{\omega\Delta t}{2}\right) && (22)\\
&= \cos\left(\omega\Delta t\right) && (23)
\end{aligned}
$$

One can also calculate this average directly by calculating the expectation value of the Hermitian operator $\frac{1}{2}\left[\sigma_x(t_j)\sigma_x(t_i)+\sigma_x(t_i)\sigma_x(t_j)\right] = \{\sigma_x(t_j),\sigma_x(t_i)\}/2$. To show this, we consider again a two level closed system evolving under the Hamiltonian $\mathcal{H} = \frac{\hbar}{2}\omega\sigma_z$ with an initial state $|\Psi(t_0)\rangle = \cos(\theta/2)|+\rangle + \sin(\theta/2)e^{i\phi}|-\rangle$. The Heisenberg picture operators and Heisenberg equation of motion for this system is

$$\sigma_x(t) = e^{i\mathcal{H}t/\hbar}\ \sigma_x\ e^{-i\mathcal{H}t/\hbar} \qquad (24)$$

$$\sigma_y(t) = e^{i\mathcal{H}t/\hbar}\ \sigma_y\ e^{-i\mathcal{H}t/\hbar} \qquad (25)$$

$$\frac{d}{dt}\sigma_x(t) = \frac{1}{i\hbar}\left[\sigma_x(t),\mathcal{H}\right] \ \Rightarrow \frac{d}{dt}\sigma_x(t) = -\omega\sigma_y(t) \qquad (26)$$

$$\frac{d}{dt}\sigma_y(t) = \frac{1}{i\hbar}\left[\sigma_y(t),\mathcal{H}\right] \ \Rightarrow \frac{d}{dt}\sigma_y(t) = \omega\sigma_x(t) \qquad (27)$$

The solutions of these coupled differential equations are given by

$$\sigma_x(t) = \sigma_x\cos(\omega t) - \sigma_y\sin(\omega t), \qquad (28a)$$

$$\sigma_y(t) = \sigma_y\cos(\omega t) + \sigma_x\sin(\omega t). \qquad (28b)$$

Using Eq.(28a), one can calculate the following expectation values

$$\langle\Psi(t_0)|\sigma_x(t_j)\sigma_x(t_i)|\Psi(t_0)\rangle = \cos\left(\omega\Delta t\right) + i\cos(\theta)\sin\left(\omega\Delta t\right) \qquad (29)$$

$$\langle\Psi(t_0)|\sigma_x(t_i)\sigma_x(t_j)|\Psi(t_0)\rangle = \cos\left(\omega\Delta t\right) - i\cos(\theta)\sin\left(\omega\Delta t\right) \qquad (30)$$

Hence, the expectation value of the Hermitian operator $\{\sigma_x(t_j),\sigma_x(t_i)\}/2$ is given by

$$
\begin{aligned}
\langle\Psi(t_0)|\{\sigma_x(t_j),\sigma_x(t_i)\}/2|\Psi(t_0)\rangle &= \langle\Psi(t_0)|\sigma_x(t_j)\sigma_x(t_i)+\sigma_x(t_i)\sigma_x(t_j)|\Psi(t_0)\rangle/2 && (31)\\
&= \cos\left(\omega\Delta t\right) && (32)
\end{aligned}
$$

where $\Delta t = (t_j - t_i)$. The expectation value given by Eq.(32) is obtained using Heisenberg equation of motion, which is exactly the same as the average value "A" calculated through probability argument given by Eq.(23).
